# Systemic immune-inflammation Index is associated with chronic kidney disease in the U.S. population: insights from NHANES 2007–2018

**DOI:** 10.3389/fimmu.2024.1331610

**Published:** 2024-02-21

**Authors:** Xiaoxin Liu, Xinyu Li, Yulin Chen, Xiaoyu Liu, Yanyan Liu, Haotian Wei, Ningxu Li

**Affiliations:** ^1^ Department of Nephrology, Liyuan Hospital, Tongji Medical College, Huazhong University of Science and Technology, Wuhan, Hubei, China; ^2^ Department of Nephrology, Tongji Hospital, Tongji Medical College, Huazhong University of Science and Technology, Wuhan, Hubei, China

**Keywords:** systemic immune inflammation index, chronic kidney disease, cross-sectional study, NHANES, population-based study

## Abstract

**Objectives:**

The systemic immune-inflammation index (SII), a novel and systematic inflammatory biomarker that is associated with chronic kidney disease (CKD), has not received much attention. This study aimed to investigate the relationship between SII and CKD in the United States (U.S.) population.

**Methods:**

Our study ultimately included a nationally representative sample of 10,787 adults who participated in the 2007-2018 National Health and Nutrition Examination Survey. Weighted multivariate logistic regression was used to assess the correlation between SII and CKD, and a restricted cubic spline (RCS) model was subsequently used to explore the non-linear relationship between SII and CKD. Subgroup analyses were performed to further the effects of other covariates on the relationship between SII and CKD.

**Results:**

Following confounder adjustment, a higher SII was related to the incidence of CKD (OR =1.36; 95% CI, 1.07–1.73; *p* =0.01), as validated by multivariable logistic regression. The RCS curve revealed a non-linear positive correlation between SII/1000 and CKD incidence (*p* for non-linear =0.0206). Additionally, subgroup analysis confirmed a stronger correlation for male participants (OR =2.628; 95% CI, 1.829-3.776) than for female participants (OR =1.733; 95% CI, 1.379-2.178) (*p* for interaction =0.046).

**Conclusions:**

SII is positively associated with the incidence of CKD among U.S. adults, especially in males. However, further studies are needed to confirm our findings and explore the causal factors that can contribute to the prevention and treatment of CKD.

## 1 Introduction

Anomalies in the structure or function of the kidneys are common patterns of chronic kidney disease (CKD) (1). The age-standardized worldwide adult CKD incidence is 11.8% for women and 10.4% for men (2). There was a 29.3% increase in the global incidence of CKD from 1990 to 2017 due to the increase in risk factors for kidney disease, including diabetes, obesity, hypertension, and the aging of the population (3). CKD contributes to substantial increases in cardiovascular mortality, all-cause mortality, cancer risk as well as cancer-related mortality (4–6) and has become a global public health problem (7). Consequently, CKD should be taken seriously.

Systemic immune-inflammation index (SII), which integrates patient platelet, lymphocyte, and neutrophil counts in peripheral blood, was recently identified as a prognostic indicator of patient inflammatory and immune status (8, 9). SII was initially utilized to assess the outcome of patients after radical resection of hepatocellular carcinoma (8). In subsequent studies, SII was linked to prognosis in patients with a multitude of cancers (e.g., colorectal, uterine, lung, and esophageal) (9–12). In addition, SII is connected to adverse outcomes in individuals with cardiovascular, respiratory, psychological, or oral diseases (13–16).

The inflammatory state is usually elevated in patients with CKD, and the systemic inflammatory response is a crucial factor in the progression of CKD (17). C-reactive protein (CRP), a classic marker of inflammation, is commonly elevated in patients (18). Several investigations have demonstrated that higher CRP levels are related to accelerated renal function decline in CKD patients (19, 20). Shankar et al. revealed a strong correlation between the likelihood of developing CKD and inflammatory biomarkers, such as white blood cell count, IL-6, and TNF-αR2 (21). Interestingly, renal tubules are home to many inflammatory cytokines and chemokines, the increase of which further accelerates the progression of kidney disease (17). Furthermore, chronic inflammation can damage the renal microvasculature to exacerbate CKD (22). In addition, the role of monocyte-to-lymphocyte ratio and platelet-to-lymphocyte ratio in predicting the prognosis of CKD has been validated (23, 24). However, there is a shortage of reports on SII in CKD patients to date and a correlation between CKD incidence and SII has not been reported.

Hence, we performed a population-based study to examine the association between SII and CKD in adult National Health and Nutrition Examination Survey (NHANES) participants. The study confirmed that SII is positively associated with the likelihood of developing CKD.

## 2 Materials and methods

### 2.1 Study population

The National Center for Health Statistics (NCHS) of the Centers for Disease Control and Prevention (CDC) annually conducts stratified multistage probability sampling known as the NHANES to track the health and nutritional conditions of the general population in the United States (U.S.) (https://www.cdc.gov/nchs/nhanes/) (25, 26). The NCHS Research Ethics Review Board authorized the NHANES in 1999, and all participants gave their notice permission before the start of the study. Every two years, survey data are made public. The NHANES website has comprehensive consent paperwork, operation instructions for surveys, and brochures for each period.

**Figure 1:**
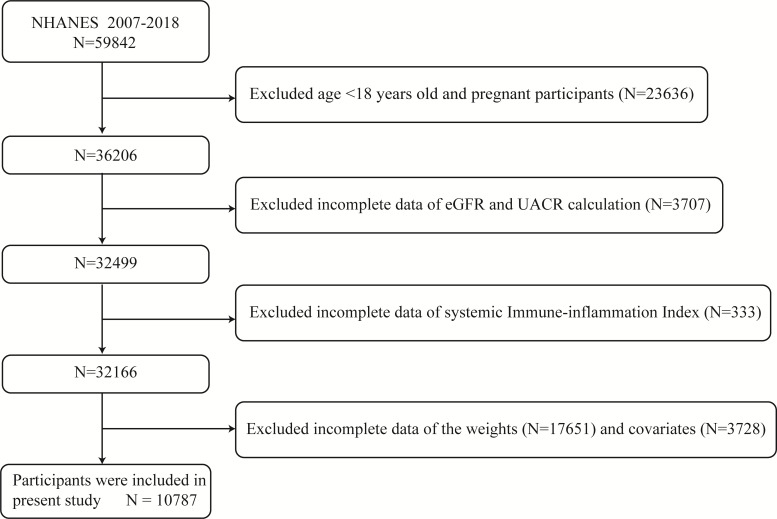
Diagram illustrating the study population’s inclusion and exclusion. NHANES, National Health and Nutrition Examination Survey; eGFR, estimated glomerular filtration rate; UACR, urinary albumin to creatinine ratio.

At mobile examination centers, standardized in-home interviews, physical exams, and laboratory tests were performed to measure the physical and nutritional health condition of the participants. The NHANES 2007–2018 database includes 59,842 total participants. Individuals who were under the age of 18 or who were pregnant, as well as those with missing data on SII, UACR, and eGFR (n =27676), unavailable data on weight values (n =17651), and no or incomplete data on covariates demanded subsequent analysis (such as alcohol status (n =1779), poverty income ratio [PIR] (n =1114), body mass index [BMI] (n =106), waist circumference [WC] (n =187), education status (n =5), smoking status (n =101), low-density lipoprotein cholesterol [LDL-C] (n =192), aspartate aminotransferase [AST] (n =18), systolic blood pressure [SBP] (n =225), serum uric acid [UA] (n= 1)) were excluded. 10,787 was the final sample size used in this analysis (**Figure 1**).

### 2.2 Data collection

#### 2.2.1 Exposure variable

SII was proved to be an exposure contributor in our study. The counts of lymphocytes, neutrophils, and platelets were calculated using automated hematology analysis equipment (a CoulterDxH 800 analyzer), and the results were reported as 10^3^ cells/mL. SII, an inflammation indicator, was calculated using the equation as follows: SII = neutrophil-to-lymphocyte ratio × platelet count.

#### 2.2.2 Outcome variable

The Jaffé rate reaction was employed to measure serum creatinine, which was subsequently converted to an estimated glomerular filtration rate (eGFR) using the established Chronic Kidney Disease Epidemiology Collaboration equation (1, 7). Solid-phase fluorescence immunoassay and a Jaffé rate reaction were utilized to assess urine albumin and creatinine. The UACR was determined using the urine albumin/creatinine ratio. The population with an eGFR less than 60 mL/min per 1.73 m^2^ or a UACR greater than or equivalent to 30 mg g^−1^ was identified as having CKD.

#### 2.2.3 Covariates

We brought the following covariates which can potentially influence the positive correlation of SII and CKD into this investigation, including age, gender, race, PIR, BMI, WC, education status, alcohol status, smoking status, DBP, SBP, diabetes mellitus(DM), hypertension, hyperlipidemia, blood glucose (Glu), alanine aminotransferase (ALT), AST, serum creatinine (Cr), UA, triglycerides (TG), total cholesterol (TC), high-density lipoprotein cholesterol (HDL-C), LDL-C, and eGFR. Measurement protocols for the above variables are detailed on www.cdc.gov/nchs/nhanes/.

### 2.3 Statistical analyses

The weighted mean ± standard deviation represents continuous data, and a weighted linear regression model was used for comparison analysis. Unweighted frequencies (weighted percentages) were used to characterize categorical variables and were analyzed using chi-square tests. The relationship between SII and CKD incidence was evaluated using multivariate logistic regression modeling. In the continuous model, the SII/1000 was used to magnify the effect value by 1000x due to the nonsignificant effect value. On the other hand, in order of lowest (Q1) to top (Q4), SII was split into quartiles, thus SII was converted to a categorical variable. Three models were used, with no covariates in Model 1; Model 2, with adjustment for age, sex, and race; and Model 3, with adjustment for age, sex, race, education status, alcohol status, smoking status, PIR, BMI, WC, DBP, SBP, Glu, ALT, AST, Cr, UA, TG, TC, HDL-C, LDL-C, eGFR, hyperlipidemia, hypertension, and DM. Furthermore, weighted restricted cubic spline (RCS) regression with three knots (10th, 50th, and 90th percentiles) was applied to analyze the potential non-linearity association between SII/1000 and the incidence of CKD. The association between SII and CKD was further analyzed and stratified by age, sex, race, BMI, hypertension, hyperlipidemia, and DM. The criteria for classifying people as normal weight, overweight, or obese were as follows: BMI less than 25, between 25 and 30, or equal to or greater than 30.0 (kg/m^2^), respectively. The pre-specified possible effect modifiers were also applied to these stratified factors. The heterogeneity of inter-subgroup associations was evaluated by interaction terms in our study. All of the statistical analyses were performed according to CDC guidelines. We reduced the significant volatility of our dataset by using a weighting strategy. We implemented all the statistical analyses in R software version 4.2.2, and *p <*0.05 was considered to indicate a statistically significant difference.

## 3 Results

### 3.1 Baseline characteristics of the participants

Of the 10,787 participants with an average age of 46.59 ± 0.30 years, 5340 were men and 5447 were women. In our research, 1789 participants (16.6%) were classified as having CKD. Within the context of CKD, marked differences (*p <*0.05) were found in groups divided by age, sex, education status, alcohol status, smoking status, hypertension, hyperlipidemia, DM, PIR, BMI, WC, SBP, DBP, Glu, AST, Cr, UA, TG, LDL-C, eGFR, and SII. By contrast, the trends for race, ALT, TC, and HDL-C were not significant (*p >*0.05). Patients with CKD were more likely to be older at diagnosis, male, fatter, and have less than a high school education, lower PIR, hypertension, hyperlipidemia, and DM, and increased SII values compared to patients without CKD. The baseline characteristics that were weighted for the research participants are displayed in **Table 1**.

**Table 1:** Weighted characteristics of the study population according to the presence or absence of chronic kidney disease.

Variable	Overall	No CKD (N=8998)	With CKD (N=1789)	*p-value*
Age, year	46.59 (0.30)	44.62 (0.30)	59.93 (0.59)	< 0.0001
Gender, %				< 0.0001
Male	5447 (50.41)	4508 (49.43)	939 (57.08)	
Female	5340 (49.59)	4490 (50.57)	850 (42.92)	
Race, %				0.09
Non-Hispanic White	4753 (68.74)	3885 (68.59)	868 (69.76)	
Non-Hispanic Black	2066 (10.50)	1710 (10.33)	356 (11.64)	
Mexican American	1642 (8.20)	1390 (8.26)	252 (7.78)	
Other Hispanic	1110 (5.33)	952 (5.46)	158 (4.47)	
Other Race	1216 (7.23)	1061 (7.36)	155 (6.36)	
Education, %				< 0.0001
Less than high school	2458 (15.11)	1937 (14.20)	521 (21.28)	
High school	2503 (22.96)	2014 (22.05)	489 (29.15)	
More than high school	5826 (61.93)	5047 (63.75)	779 (49.56)	
Alcohol status, %				< 0.0001
Never	1512 (10.79)	1194 (10.06)	318 (15.70)	
Former	1597 (12.07)	1195 (10.92)	402 (19.83)	
Mild	3777 (37.84)	3161 (37.96)	616 (37.03)	
Moderate	1679 (17.85)	1492 (18.60)	187 (12.79)	
Heavy	2222 (21.45)	1956 (22.45)	266 (14.66)	
Smoking status, %				< 0.0001
Never	6079 (56.31)	5150 (56.93)	929 (52.16)	
Former	2601 (24.89)	2048 (23.93)	553 (31.39)	
Now	2107 (18.80)	1800 (19.15)	307 (16.44)	
PIR	3.02 (0.04)	3.07 (0.04)	2.68 (0.07)	< 0.0001
Hypertension, %				< 0.0001
No	6351 (63.36)	5846 (67.99)	505 (31.95)	
Yes	4436 (36.64)	3152 (32.01)	1284 (68.05)	
Hyperlipidemia, %				< 0.0001
No	3147 (30.58)	2837 (32.30)	310 (18.91)	
Yes	7640 (69.42)	6161 (67.70)	1479 (81.09)	
Diabetes mellitus, %				< 0.0001
No	6627 (67.40)	6006 (71.40)	621 (40.31)	
Pre-diabetes mellitus	1974 (17.58)	1597 (16.98)	377 (21.70)	
Diabetes mellitus	2186 (15.01)	1395 (11.63)	791 (37.99)	
BMI, kg/m^2^	28.89 (0.11)	28.74 (0.11)	29.90 (0.27)	< 0.0001
WC, cm	98.98 (0.27)	98.37 (0.28)	103.09 (0.63)	< 0.0001
Systolic blood pressure, mmg	120.68 (0.25)	119.22 (0.26)	130.53 (0.57)	< 0.0001
Diastolic blood pressure, mmg	69.77 (0.23)	69.97 (0.24)	68.41 (0.45)	< 0.001
Blood glucose, uIU/ml	5.87 (0.02)	5.75 (0.02)	6.65 (0.07)	< 0.0001
Alanine aminotransferase, IU/L	25.01 (0.20)	25.15 (0.21)	24.04 (0.57)	0.07
Aspartate aminotransferase, IU/L	24.99 (0.19)	24.79 (0.19)	26.37 (0.70)	0.03
Serum creatinine, mg/dl	77.26 (0.33)	74.37 (0.23)	96.85 (2.10)	< 0.0001
Serum uric acid, mmol/L	326.21 (1.18)	321.60 (1.20)	357.46 (2.44)	< 0.0001
Triglycerides, mmol/L	1.29 (0.01)	1.27 (0.01)	1.43 (0.03)	< 0.0001
Total cholesterol, mmol/L	4.94 (0.02)	4.94 (0.02)	4.91 (0.03)	0.41
HDL-C, mmol/L	1.41 (0.01)	1.40 (0.01)	1.42 (0.02)	0.42
LDL-C, mmol/L	2.94 (0.01)	2.95 (0.01)	2.84 (0.03)	< 0.001
eGFR, ml/min/1.73m^2^	95.91 (0.39)	98.91 (0.37)	75.62 (1.00)	< 0.0001
Systemic immune-inflammation index	511.77 (4.27)	500.38 (4.24)	589.05 (12.63)	< 0.0001

Mean ± SD for continuous variables, % for categorical variables. PIR poverty income ratio; BMI body mass index; WC waist circumference; HDL-C high-density lipoprotein cholesterol; LDL-C low-density lipoprotein cholesterol; eGFR estimated glomerular filtration; CKD chronic kidney disease.

### 3.2 Association between SII and CKD

**Figure 2:**
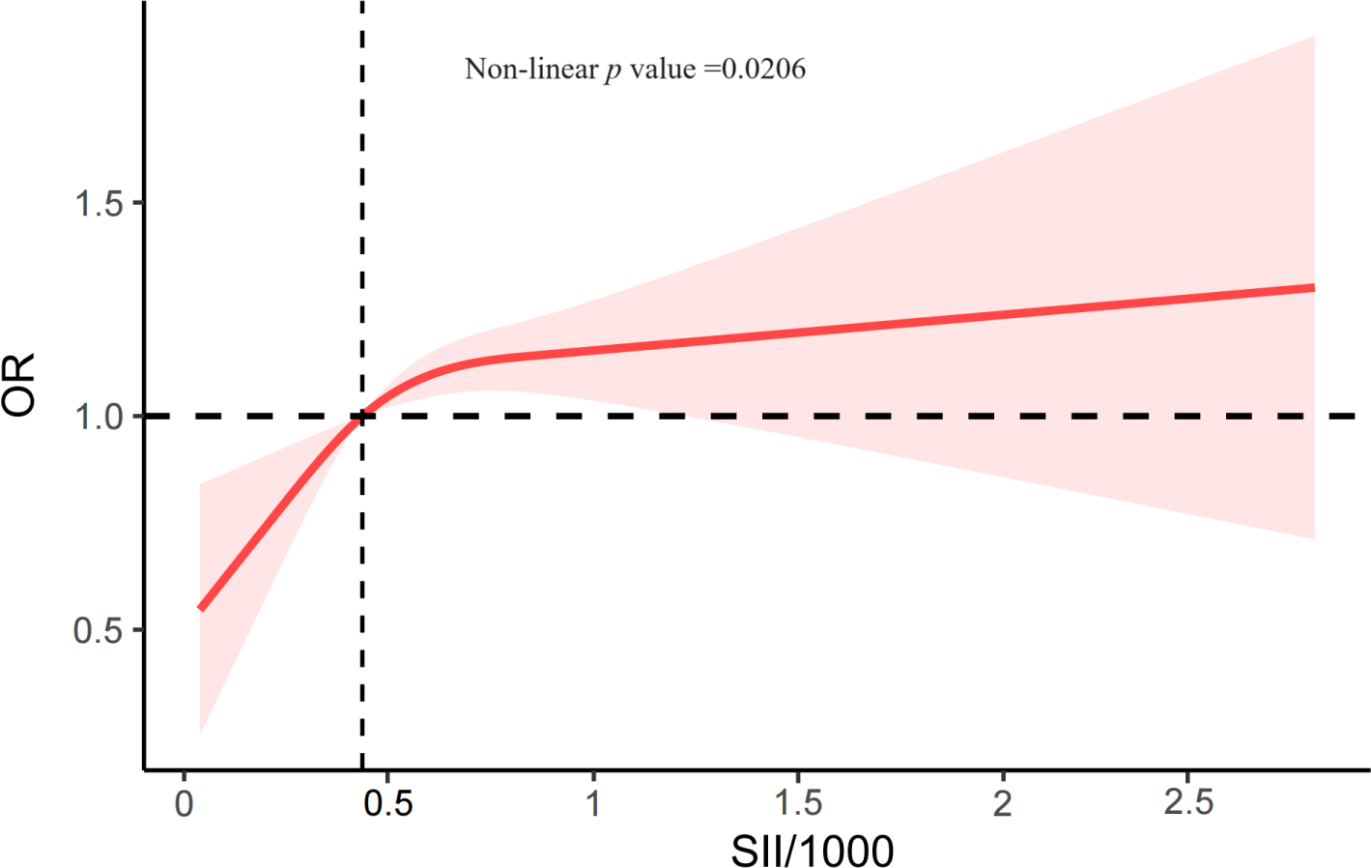
The RCS plot describes the association between SII/1000 and the incidence of CKD. It was adjusted for all covariates in **Table 3**. SII, systemic immune-inflammation index; OR, odds ratio.

The associations between CKD and several factors, including history of DM, hyperlipidemia and hypertension, age, sex, race, education, PIR, alcohol status and smoking status, BMI, WC, SBP, and DBP, several biochemical markers, and SII/1000, are shown in **Table 2**. According to our research, a rise in SII was linked to higher CKD. Three models were developed to identify the connection between SII and the incidence of CKD in this study (**Table 3**). According to Model 1, the odds ratio (OR) and 95% confidence interval (CI) were 2.14 (1.75–2.62; *p <*0.0001), which suggested that the incidence of CKD with each unit increase in SII/1000. Similar trends were also demonstrated in Model 2 (OR [95% CI], 1.77 [1.43–2.21]; *p <*0.0001) and Model 3 (OR [95% CI], 1.36 [1.07– 1.73]; *p* =0.01). Sensitivity analysis was performed for the SII quartiles, and participants in Quartile 4 and Quartile 3 had 34% and 27% the incidence of CKD, respectively, than did those in Quartile 1 (OR =1.34; 95% CI, 1.04–1.71, *p* =0.02) and (OR =1.27; 95% CI, 1.02–1.59, *p* =0.03) in Model 3. Furthermore, participants in Quartile 2 demonstrated the incidence of CKD than did those in Quartile 1, but such a connection was not statistically significant according to Model 3 (OR =1.20; 95% CI, 0.93-1.56; *p* =0.16). In addition, the RCS was used to assess the nonlinear relationship between SII/1000 and CKD. We found a nonlinear positive correlation between these variables (non-linear *p* value =0.0206), with the OR curve for CKD first rising sharply as SII increased and then leveling off (**Figure 2**).

**Table 2:** Univariate logistic regression models of CKD.

Variables	OR^1^ (95% CI^2^)	*p-value*
Age, year	1.06 (1.05,1.07)	<0.0001
Female (versus male)	0.74 (0.65,0.83)	<0.0001
Race (versus Non-Hispanic White)		
Non-Hispanic Black	1.11 (0.94,1.30)	0.22
Mexican American	0.93 (0.77,1.12)	0.42
Other Hispanic	0.81 (0.62,1.04)	0.10
Other Race	0.85 (0.69,1.05)	0.13
Education status (versus less than high school)		
High school	0.88 (0.73,1.07)	0.19
More than high school	0.52 (0.42,0.63)	<0.0001
Alcohol status (versus never)		
Former	1.16 (0.92,1.48)	0.21
Mild	0.63 (0.50,0.79)	<0.001
Moderate	0.44 (0.33,0.59)	<0.0001
Heavy	0.42 (0.33,0.54)	<0.0001
Smoking status (versus never)		
Former	1.43 (1.22,1.68)	<0.0001
Now	0.94 (0.77,1.14)	0.51
PIR	0.87 (0.83,0.91)	<0.0001
Hypertension (yes versus no)	4.53 (3.90,5.26)	<0.0001
Hyperlipidemia (yes versus no)	2.05 (1.70,2.45)	<0.0001
Diabetes mellitus (versus no)		
Pre-diabetes mellitus	2.26 (1.87,2.74)	<0.0001
Diabetes mellitus	5.79 (4.88,6.87)	<0.0001
BMI, kg/m^2^	1.02 (1.01,1.03)	<0.0001
WC, cm	1.02 (1.01,1.02)	<0.0001
Systolic blood pressur, mmg	1.04 (1.03,1.04)	<0.0001
Diastolic blood pressure, mmg	0.99 (0.98,0.99)	<0.001
Blood Glucose, uIU/ml	1.27 (1.23,1.32)	<0.0001
Alanine aminotransferase, IU/L	1.00 (0.99,1.00)	0.12
Aspartate aminotransferase, IU/L	1.00 (1.00,1.01)	0.05
Serum creatinine, mg/dl	1.04 (1.04,1.04)	<0.0001
Serum uric acid, mmol/L	1.01 (1.00,1.01)	<0.0001
Triglycerides, mmol/L	1.29 (1.18,1.41)	<0.0001
Total cholesterol, mmol/L	0.97 (0.90,1.04)	0.41
HDL-C, mmol/L	1.08 (0.90,1.28)	0.41
LDL-C, mmol/L	0.86 (0.79,0.94)	<0.001
eGFR, ml/min/1.73m^2^	0.95 (0.95,0.95)	<0.0001
SII/1000	2.14 (1.75,2.62)	<0.0001

Next to the variables are the unit for continuous variables and the reference group for categorical variables. PIR poverty income ratio; BMI body mass index; WC waist circumference; HDL-C high-density lipoprotein cholesterol; LDL-C low-density lipoprotein cholesterol; eGFR estimated glomerular filtration; SII, systemic immune-inflammation index. ^1^OR, odds ratio; ^2^CI, confidence interval.

**Table 3:** Multivariate logistic regression of systemic immune-inflammation index for CKD.

OR^1^ (95%CI^2^), *p-value*			
	Model 1	Model 2	Model 3
SII/1000	2.14 (1.75, 2.62), <0.0001	1.77 (1.43, 2.21), <0.0001	1.36 (1.07, 1.73), 0.01
SII quartiles			
Q1	Ref.	Ref.	Ref.
Q2	1.19 (0.96, 1.48), 0.11	1.26 (1.01, 1.56), 0.04	1.20 (0.93, 1.56), 0.16
Q3	1.42 (1.17, 1.71), <0.001	1.35 (1.11, 1.64), 0.003	1.27 (1.02, 1.59), 0.03
Q4	1.84 (1.52, 2.24), <0.0001	1.66 (1.36, 2.02), <0.0001	1.34 (1.04, 1.71), 0.02

Model 1: unadjusted. Model 2: adjusted by age, gender, and race. Model 3: adjusted by age, gender, race, education status, alcohol status, smoking status, PIR, BMI, WC, DBP, SBP, Glu, ALT, AST, Cr, UA, TG, TC, HDL-C, LDL-C, eGFR, hyperlipidemia, hypertension, diabetes mellitus; SII, systemic immune-inflammation index; ^1^OR, odds ratio; ^2^CI, confidence interval.

### 3.3 Subgroup analysis

**Figure 3:**
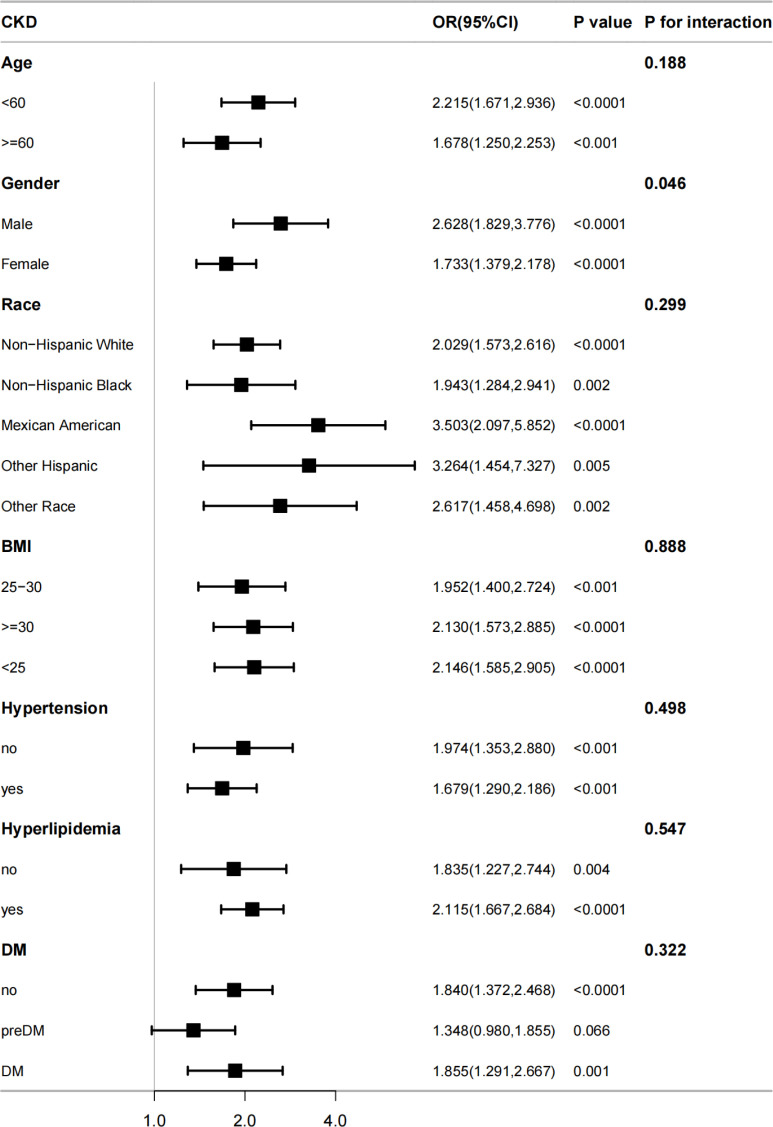
The association between SII and CKD by different subgroups. BMI, body mass index; DM, diabetes mellitus; CKD, chronic kidney disease; OR, odds ratio; CI, confidence interval.

To determine whether there is a substantial correlation between SII and CKD in particular subpopulations, our study also employed subgroup analysis (**Figure 3**). The participants were initially classified by age, sex, race, BMI, hypertension, hyperlipidemia, and DM. These subgroups were subsequently subjected to another round of logistic regression model analysis. All covariates in **Table 3**, except those used for stratification, were adjusted in the model. In all the subgroups stratified by age, sex, race, BMI, hypertension, and hyperlipidemia, SII was significantly correlated with CKD. Nonetheless, there was also a positive, albeit not statistically significant, connection between SII and CKD in individuals with pre-DM (OR =1.348; 95% CI, 0.980-1.855; *p* =0.066). Age, race, BMI, hypertension, hyperlipidemia, and DM did not significantly affect the correlation between SII and CKD according to interaction tests (*p >*0.05 for all interactions). Nevertheless, we found notable interactions in the gender subgroups. There was a greater correlation between SII and CKD in male individuals (OR =2.628) than in female individuals (OR =1.733).

## 4 Discussion

It is the first study that focused on the connection between SII and the likelihood of getting CKD. We investigated the relationship in a nationally representative sample of the U.S. adult population. After adjusting for multiple covariates, we detected that SII was positively correlated with the likelihood of getting CKD via multivariate logistic regression analysis. Furthermore, the RCS curve showed that SII/1000 was positively and non-linearly correlated with the prevalence of CKD. Eventually, both the subgroup analysis and interaction tests revealed that the association between SII and CKD was significant in males.

According to the Global Burden of Disease studies, CKD has become a major global cause of mortality (27, 28). A growing amount of research has found that markers of inflammation are strongly linked with the development of CKD and the prognosis of CKD patients, however, there is still a gap in research on the connection between SII and CKD (29–31). An Urban Setting (HELIUS) cohort study enrolling a total of 5,740 adult participants found that low-grade inflammatory biomarkers (fibrinogen and D-dimer) were significantly linked with the risk of developing CKD and that there were significant differences between races (29). Lousa et al. (30) demonstrated that circulating levels of inflammatory molecules, CRP, interleukin-6, tumor necrosis factor-α, tumor necrosis factor receptor 2, and leptin were significantly negatively correlated with eGFR, and interestingly, TNFR2 increased steadily with increasing stage in CKD patients. A community-based, prospective cohort with 8,057 non-CKD participants in Korea suggested that a low serum albumin/globulin was one of the independent indicators of CKD progression (31). Consistent with previous studies, we also confirm a positive connection between the level of inflammation and the risk of developing CKD.

Numerous pathophysiologic alterations are responsible for the development of CKD, and immunological dysfunction and elevated inflammation may be key players in this process (32). SII is a method that quantifies the interaction between systemic inflammation and immune response (8). SII was calculated from peripheral blood neutrophil, platelet, and lymphocyte counts. This means that SII is more readily available in clinical practice compared to other indicators of inflammation and can reduce the financial burden on patients. Most importantly, SII has been shown in many studies to be more responsive to the body’s inflammatory state and to have greater prognostic value than other inflammatory markers (8, 9, 33, 34). As previously described, SII was initially used primarily in oncology patients, however, with further research it also is strongly associated with other diseases such as osteoporosis among middle-aged and older people (35), ulcerative colitis (36), metabolic syndrome (37), heart failure (38), periodontitis (16), chronic obstructive pulmonary disease (14), coronary heart disease (39), hyperlipidemia (40), cognitive impairment (41), rheumatoid arthritis (42) and depression (15).

Recently, considerable studies have been invested in exploring the link between SII and renal illness (33, 43–45). A large multi−center longitudinal study with non-dialysis dependent CKD undergoing coronary angiography patients in China suggested that elevated SII levels at hospital admission in CKD patients are an independent risk element for their all-cause mortality (45). Di et al. reported that SII is positively connected with a high risk of kidney stones in U.S. adults under 50 years old (44). Furthermore, in patients with severe acute pancreatitis (SAP), Lu et al. discovered SII to be a new, straightforward, and reliable marker for early prediction of acute kidney damage (33). Interestingly, A Population-Based Study from the NHANES (2011–2018) suggested that compared to patients without DKD, type 2 diabetes (T2DM) patients with DKD had considerably higher SII levels, and SII levels were linked to a greater risk of DKD in T2DM patients (43). No research, however, has been devoted to exploring the connection between SII and the incidence of suffering from CKD.

Specific mechanisms linking inflammation and CKD are unclear. There is now significant evidence that an inflammatory response of any cause can cause renal injury, thereby promoting the initiation and progression of CKD (22). A study found a decrease in caspase-1 activation and IL-1β and IL-18 maturation after UUO (unilateral urethral obstruction) in NLRP3-/- mice thereby attenuating tubular injury, inflammation, and fibrosis (46). Furthermore, the consequences of CKD, including sepsis, fibrosis, and accelerated vascular calcification, may be influenced by the IL-1β/IL-18 axis (22). In addition, TNF-α-/- mice on a high-fat diet attenuate glomerulosclerosis and renal fibrosis by reducing glomerular oxidative stress (47).

Although neutrophils, platelets, and lymphocytes serve as simple indicators of the inflammatory response, they have a great impact on the mechanisms underlying the development of CKD. Several investigations have demonstrated that the death of renal cells results in the production of damage-associated molecular patterns (DAMPs), which in turn cause innate immune cells—primarily neutrophils and macrophages—to infiltrate the area (48, 49). Neutrophils can directly injure renal cells by releasing reactive oxygen species (ROS) and granular substances, or they can indirectly damage renal cells by activating other immune cells through the production of inflammatory mediators (50, 51). According to Ryu et al., the Siglec-F^+^ neutrophils are essential for fostering a pro-fibrotic microenvironment in renal fibrosis (52). Siglece-F^+^ neutrophils can release collagen 1 and generate pro-fibrotic cytokines, both of which can lead to renal fibrosis, strikingly, when Siglece-F^+^ neutrophils are increased after the onset of CKD, progressive renal fibrosis is markedly exacerbated (52). Additionally, significant participants in inflammation are platelets (53). In a mouse model of antibody-mediated chronic kidney disease (AMCKD), renal-derived thrombopoietin (TPO) boosts the production of myeloid cells and platelets, aggravating chronic thromobinflammation in the microvasculature, and TPO neutralization can ameliorate this kidney disease (54). Several types of lymphocytes and the mediators they produce can reduce renal inflammation and are protective against renal fibrosis (55, 56). When kidney damage occurs, tissue-resident IL-33R+ and IL-2Ra+ regulatory T cells protect against fibrosis (57). Furthermore, IFN-γ-producing CD8^+^ T cells prevent CD4^+^ T cells from differentiating into Th2 cells, which reduces renal inflammation and fibrosis, and fibroblast apoptosis may be triggered by CD11c^+^CD8^+^ T cells in obstructed kidney (56).

The present study has the following strengths. First, we used a large, nationally representative sample of U.S. adults (n = 10787). Second, throughout the study, we improved the reliability of the results by using appropriate weights and adjusting for existing and potentially confounding factors affecting the onset of CKD. In addition, we investigated the possible non-linear relationship between SII and CKD risk by RCS analysis. This study has several limitations. First, it was not possible to obtain causality due to the inherent nature of the cross-sectional study design. Second, the relationship between SII and CKD may be affected by residual confounders even after potential confounders have been taken into account. Third, since the population in this study included adult Americans and did not include minors or people from other countries, additional research is needed to determine whether the connection between SII and CKD should be extended to other groups.

## 5 Conclusion

Our findings suggest that higher SII values are associated with the incidence of CKD, and this influence is more noticeable in the male population. Notably, to further validate these results, further cohort studies or randomized controlled trials are urgently needed. Furthermore, the underlying mechanisms need to be clarified as soon as possible to provide additional means of preventing CKD.

## Data availability statement

The original contributions presented in the study are included in the article/supplementary material. Further inquiries can be directed to the corresponding authors.

## Ethics statement

The studies involving humans were approved by NCHS Ethics Review Board (ERB). The studies were conducted in accordance with the local legislation and institutional requirements. The participants provided their written informed consent to participate in this study.

## Author contributions

XXL: Writing – original draft, Writing – review & editing. XinL: Investigation, Writing – review & editing. YC: Writing – review & editing. XiaL: Writing – review & editing. YL: Supervision, Writing – review & editing. HW: Software, Supervision, Writing – review & editing. NL: Supervision, Writing – original draft, Writing – review & editing.
